# Supplementary material for: Functional Characterization of JcSWEET12 and JcSWEET17a from Physic Nut
Source: Int J Mol Sci. 2024 Jul 26;25(15):8183. doi: 10.3390/ijms25158183 (PMC11311823; doi:10.3390/ijms25158183)
Supplement: Supplementary file 1 [file ijms-25-08183-s001.zip › Table S1 Primers used in this study..pdf]

Table S1 Primers used in this study.

Name	Sequences（5'-3'）	length （bp）	restriction enzyme
**Gene cloning**			
JcSWEET12-F	-GGCgagctcTCCTTGTTGTTTTGAGAG-	910	*Sac* I
JcSWEET12-R	-GGCtctagaATCAAGCATGACATGCAA-		*Xba* I
JcSWEET17a-F	-GGCgagctcCTATCTTCAGTAAAACAT-	757	*Sac* I
JcSWEET17a -R	-CGAActgcagTTATGGGTTTTCCCGAGG-		*Pst* I
**Subcellular localization**			
JcSWEET12-F	-CGGggtaccATGGGCTTGTTTTCCACT-	870	*Kpn* I
JcSWEET12-R	-CGCgtcgacAGCATGACATGCAATAAG-		*Sal* I
JcSWEET17a-F	-ggtaccATGGAAGGTTTAATCTTG-	717	*Kpn* I
JcSWEET17a -R	-gtcgacTGGGTTTTCCCGAGGTGT-		*Sal* I
**Overexpression in Arabidopsis**			
JcSWEET12-F	-CGGggtaccATGGGCTTGTTTTCCACT-	873	*Kpn* I
JcSWEET12-R	-CGCgtcgacTCAAGCATGACATGCAATAAG-		*Sal* I
JcSWEET17a-F	-ggtaccATGGAAGGTTTAATCTTG-	720	*Kpn* I
JcSWEET17a -R	-gtcgacTCATGGGTTTTCCCGAGGTGT-		*Sal* I
**Semi-quantitative RT-PCR**			
SWEET12-F	-TCAACAGAAGGGTTCCAATC-	497	
SWEET12-R	-ACCCCACATAGAAATCCCAA-		
JcSWEET17a-F	-CAGATCGACGGAGGATTTTG-	375	
JcSWEET17a -R	-TCCACGCTTTTCGTTGTCAC-		
AtActin (AT3G18780)-F	-AGATGCCCAGAAGTCTTGTTCC-	180	
AtActin (AT3G18780) -R	-TTTGCTCATACGGTCAGCGATA-		
