# Supplementary figures and images for: Functional Characterization of JcSWEET12 and JcSWEET17a from Physic Nut
Source: Int J Mol Sci. 2024 Jul 26;25(15):8183. doi: 10.3390/ijms25158183 (PMC11311823; doi:10.3390/ijms25158183)

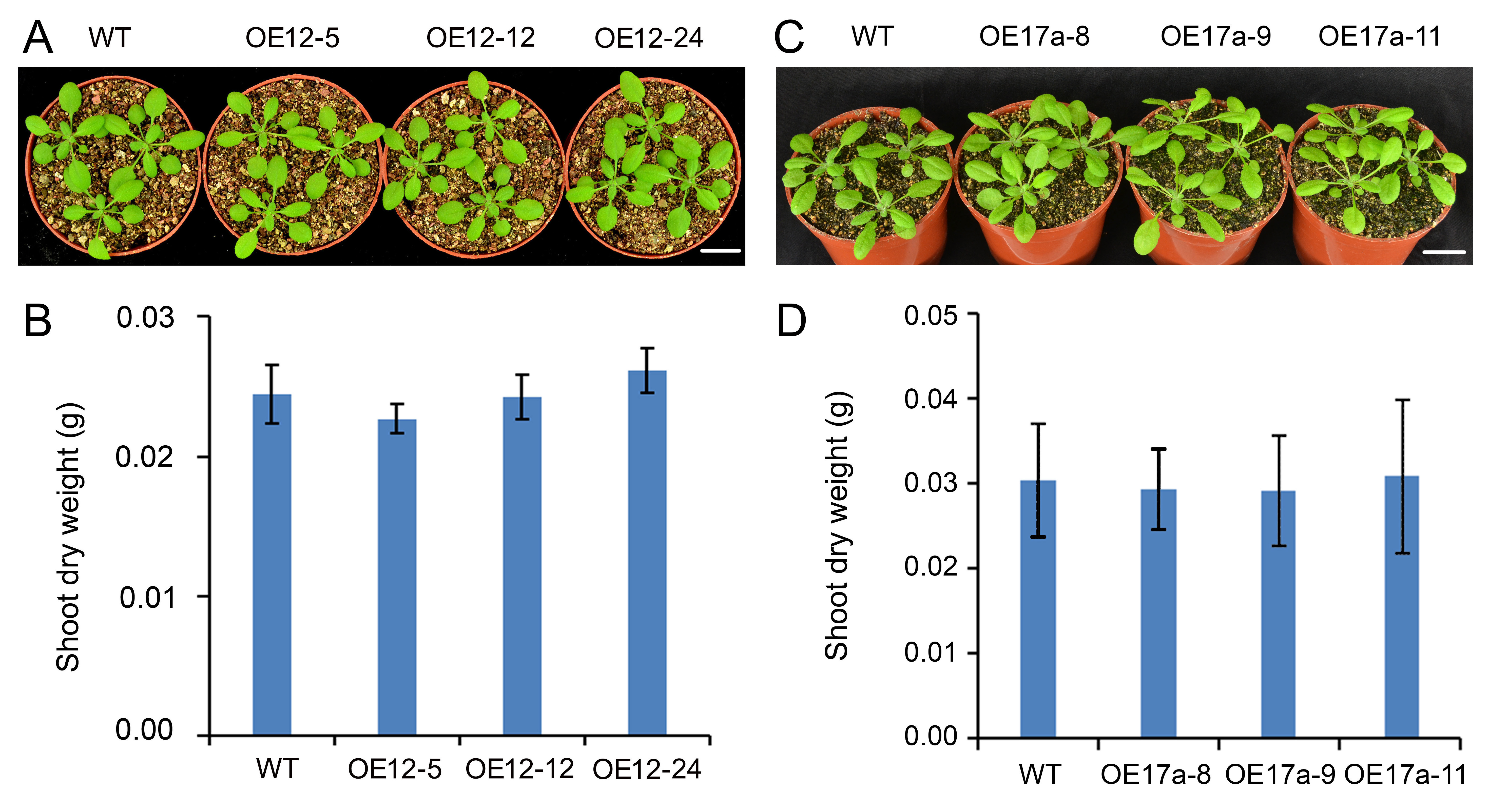

Supplement: Supplementary file 1 [file ijms-25-08183-s001.zip › Figure S1.jpg]

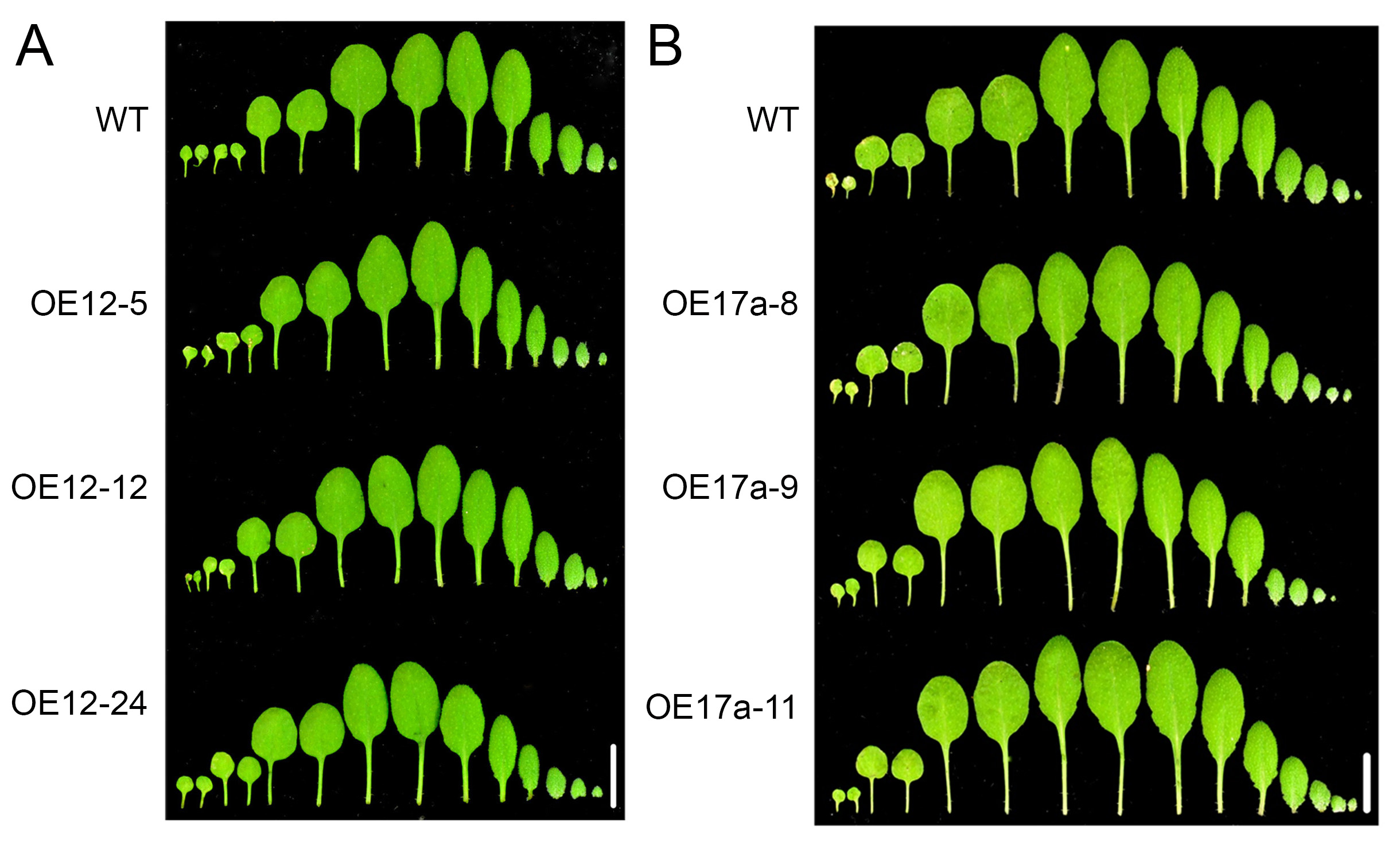

Supplement: Supplementary file 1 [file ijms-25-08183-s001.zip › Figure S2.jpg]

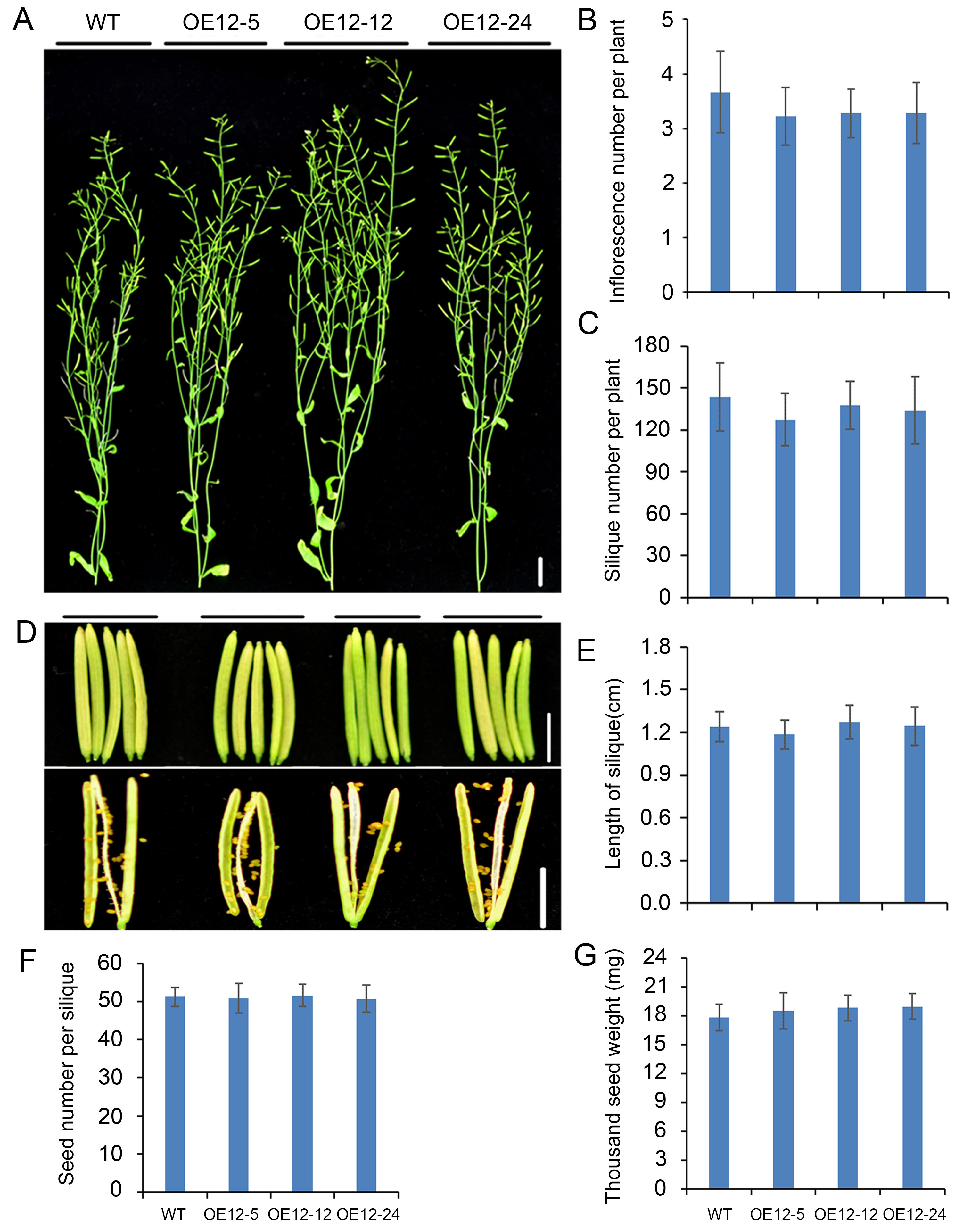

Supplement: Supplementary file 1 [file ijms-25-08183-s001.zip › Figure S3.jpg]

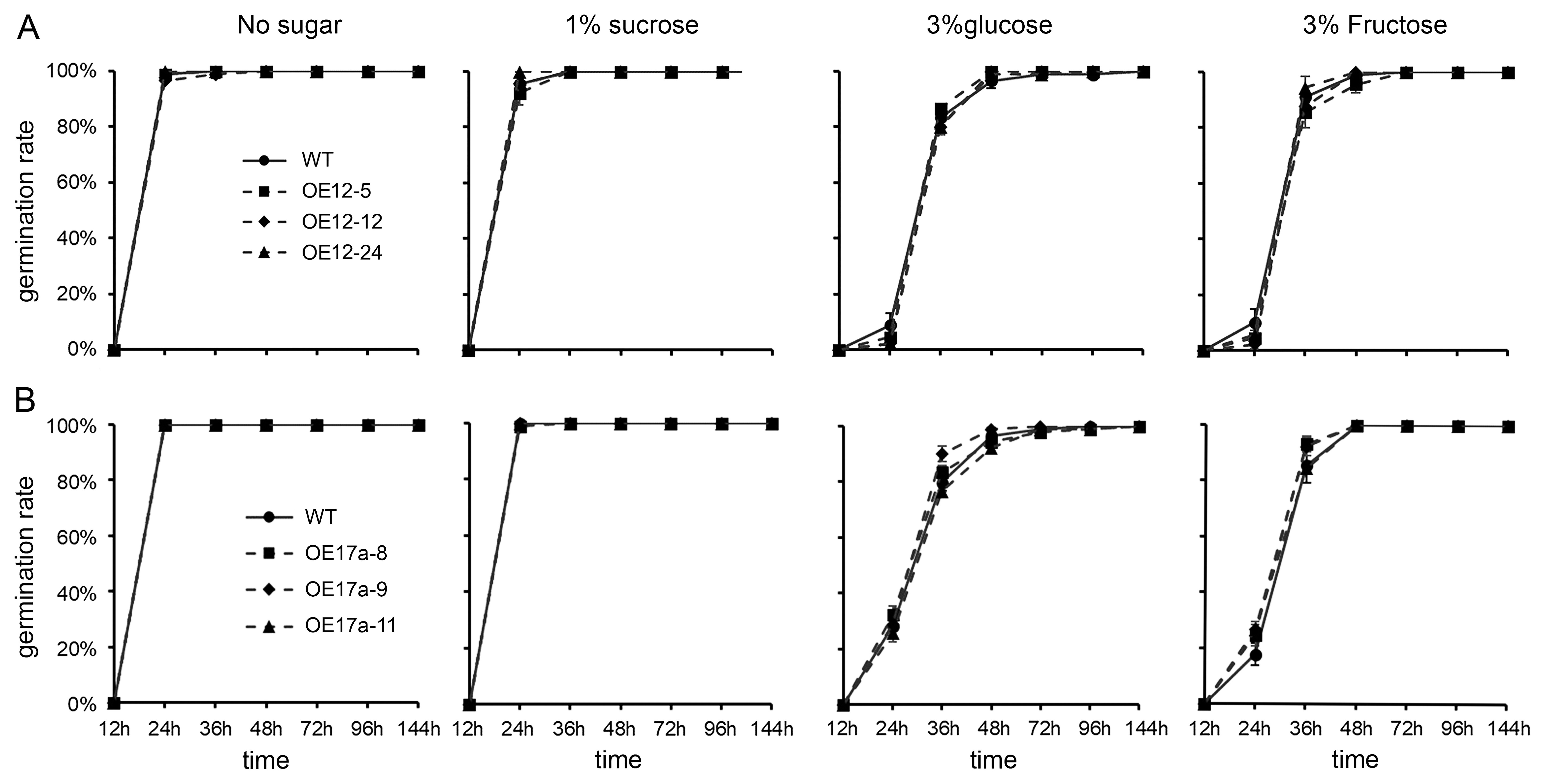

Supplement: Supplementary file 1 [file ijms-25-08183-s001.zip › Figure S4.jpg]

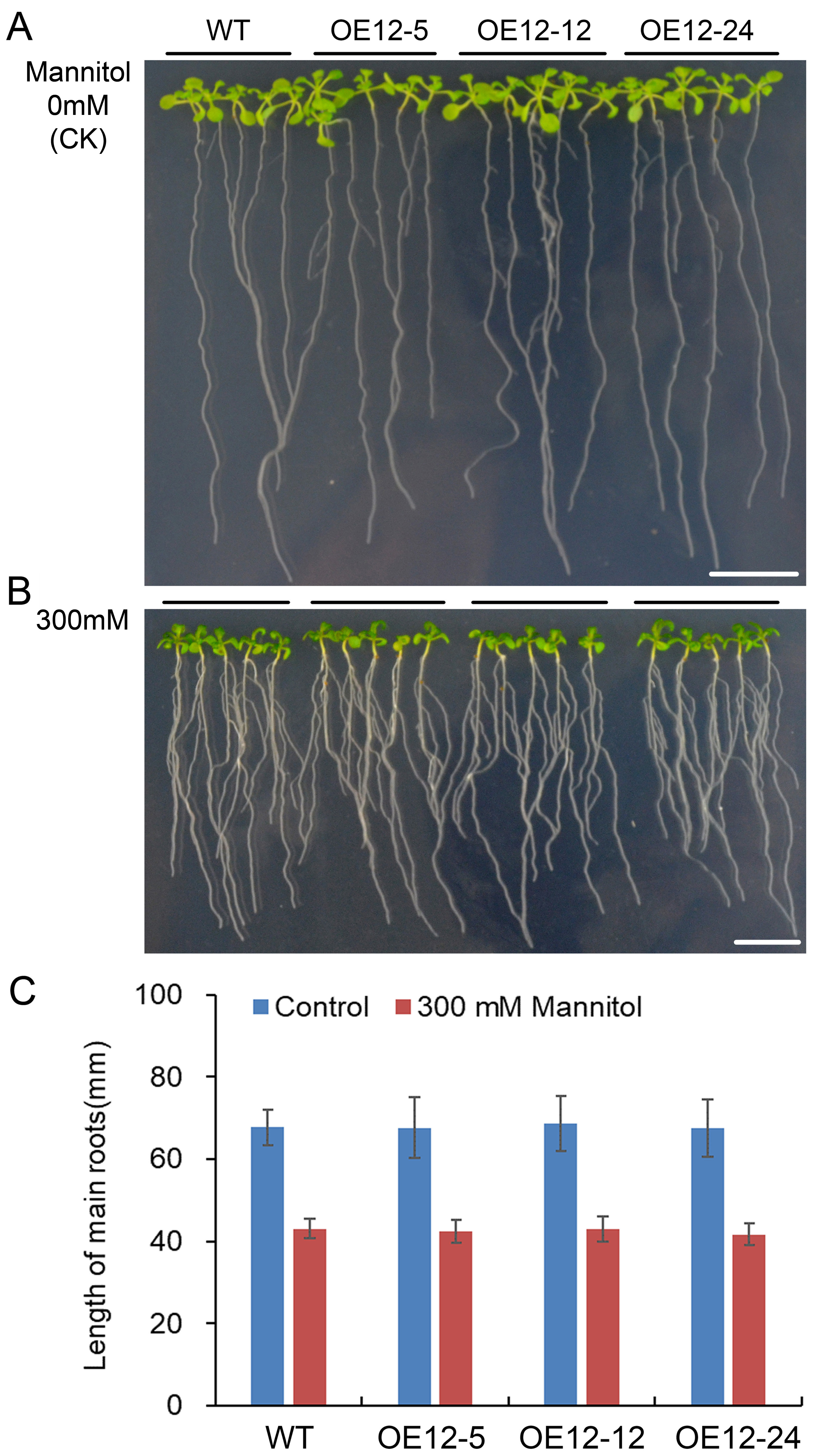

Supplement: Supplementary file 1 [file ijms-25-08183-s001.zip › Figure S5.jpg]

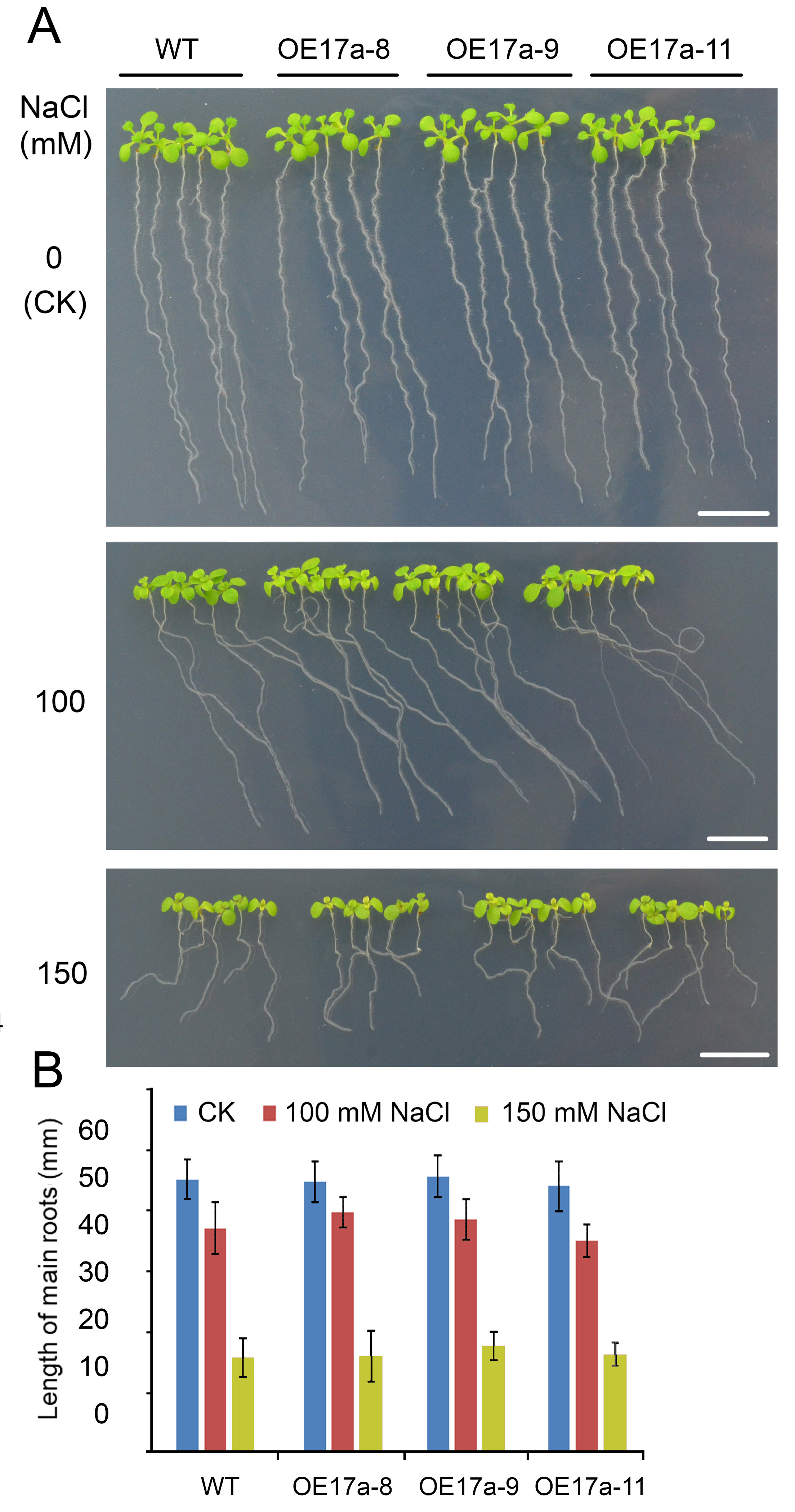

Supplement: Supplementary file 1 [file ijms-25-08183-s001.zip › Figure S6.jpg]

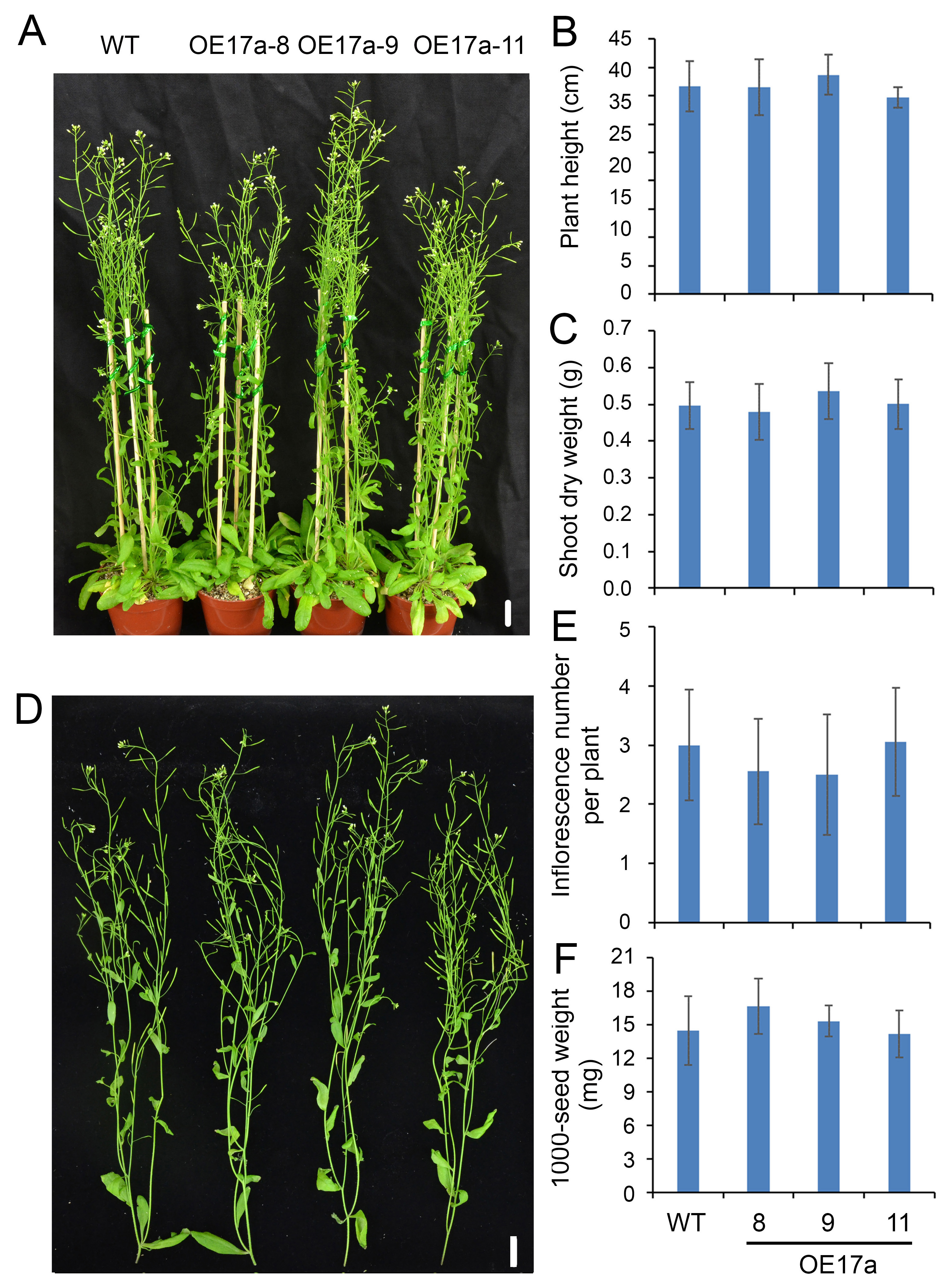

Supplement: Supplementary file 1 [file ijms-25-08183-s001.zip › Figure S7.jpg]
